# Metapopulation Vicariance, Age of Island Taxa and Dispersal: A Case Study Using the Pacific Plant Genus *Planchonella* (Sapotaceae)

**DOI:** 10.1093/sysbio/syz025

**Published:** 2019-04-23

**Authors:** Ulf Swenson, J Christopher Havran, Jérôme Munzinger, Stephen Mcloughlin, Stephan Nylinder

**Affiliations:** 1 Department of Botany, Swedish Museum of Natural History, Box 50007, SE-104 05 Stockholm, Sweden; 2 Department of Biological Sciences, Campbell University, 205 Day Dorm Road, Buies Creek, NC 27506, USA; 3 AMAP, IRD, CIRAD, CNRS, INRA, Université Montpellier, 34000 Montpellier, France; 4 Department of Palaeobiology, Swedish Museum of Natural History, Box 50007, SE-104 05 Stockholm, Sweden; 5 Department of Psychology, Gothenburg University, Box 500, SE-405 30 Göteborg, Sweden

**Keywords:** Dispersal, divergence times, Fiji, Hawaii, metapopulation vicariance, molecular dating, Society Islands, Vanuatu

## Abstract

Oceanic islands originate from volcanism or tectonic activity without connections to continental landmasses, are colonized by organisms, and eventually vanish due to erosion and subsidence. Colonization of oceanic islands occurs through long-distance dispersals (LDDs) or metapopulation vicariance, the latter resulting in lineages being older than the islands they inhabit. If metapopulation vicariance is valid, island ages cannot be reliably used to provide maximum age constraints for molecular dating. We explore the relationships between the ages of members of a widespread plant genus (*Planchonella*, Sapotaceae) and their host islands across the Pacific to test various assumptions of dispersal and metapopulation vicariance. We sampled three nuclear DNA markers from 156 accessions representing some 100 Sapotaceae taxa, and analyzed these in BEAST with a relaxed clock to estimate divergence times and with a phylogeographic diffusion model to estimate range expansions over time. The phylogeny was calibrated with a secondary point (the root) and fossils from New Zealand. The dated phylogeny reveals that the ages of *Planchonella* species are, in most cases, consistent with the ages of the islands they inhabit. *Planchonella* is inferred to have originated in the Sahul Shelf region, to which it back-dispersed multiple times. Fiji has been an important source for range expansion in the Pacific for the past 23 myr. Our analyses reject metapopulation vicariance in all cases tested, including between oceanic islands, evolution of an endemic Fiji–Vanuatu flora, and westward rollback vicariance between Vanuatu and the Loyalty Islands. Repeated dispersal is the only mechanism able to explain the empirical data. The longest (8900 km) identified dispersal is between Palau in the Pacific and the Seychelles in the Indian Ocean, estimated at 2.2 Ma (0.4–4.8 Ma). The first split in a Hawaiian lineage (*P. sandwicensis*) matches the age of Necker Island (11.0 Ma), when its ancestor diverged into two species that are distinguished by purple and yellow fruits. Subsequent establishment across the Hawaiian archipelago supports, in part, progression rule colonization. In summary, we found no explanatory power in metapopulation vicariance and conclude that *Planchonella* has expanded its range across the Pacific by LDD. We contend that this will be seen in many other groups when analyzed in detail.

Colonization of oceanic islands is usually perceived as a process of long-distance dispersal (LDD) across open waters from continents or other islands with or without subsequent founder events (Carlquist 1974; Whittaker et al. 2017). The process can be explained in terms of island progression rule colonization, involving dispersals linked to the appearance and development of new islands. This process stipulates that older islands should harbor older lineages than younger islands, a relationship that is proposed to be useful for providing maximum age bounds for calibrating molecular date estimates when fossils are scarce or absent (Ho and Philipps 2009; Nattier et al. 2011; Woo et al. 2011; Swenson et al. 2014; Ho et al. 2015).

Metapopulation vicariance is an alternative explanation of how organisms colonize oceanic islands (Heads 2006, 2009, 2011, 2017, 2018). The idea is in stark contrast to and rejects LDD and founder speciation. Instead, a concept of “normal” or “local” ecological dispersal is invoked, a type of “short dispersal” that results in species’ range expansion across archipelagoes without speciation. In other words, a species disperses from one island to another by local ecological dispersal without a founder effect and without evolving into a new species that differs from the ancestor and, instead, survives as an old species in a dynamic environment. Subsequent volcanic activities or plate movements will eventually separate populations so that vicariant species may evolve. The age of such a sister pair is younger than or approximately the same as the islands they inhabit, but since species form metapopulations and move to younger islands by local ecological dispersals, the reconstructed divergence events are older than the land inhabited by these species. Hence, this process putatively accounts for the distribution of unique species or, according to Heads (2018), even clades that are older than the island they inhabit. In metapopulation vicariance, island age is irrelevant because species on islands can be much older than the islands they inhabit, and therefore, it is argued to be inappropriate to use island age as a maximum age constraint in molecular dating analyses (Heads 2011; Pillon and Buerki 2017). Several examples of metapopulation vicariance come from the Pacific Ocean (Heads 2005, 2008, 2011), possibly best exemplified by the plant genus *Coprosma* (Rubiaceae: Heads 2017) (but see Cantley et al. (2016) for an alternative interpretation of the generic history). Criteria to distinguish between LDD and local ecological dispersal have never been formulated; but Heads (2017, p. 423) has attempted to identify differences between the two mechanisms.

The origins of the Fiji and Vanuatu biotas have been less studied than those of other regions in the southwest Pacific. With their strong affinities to Australia, New Caledonia, and Malesia, especially New Guinea, these floras are generally interpreted to be the result of LDD (Smith 1979; Keppel et al. 2009). Fiji and Vanuatu have complex geological histories, summarized by Hall (2002) and Crawford et al. (2003), with landmasses initially developing as an island arc system (the Vitiaz Arc) incorporating the older parts of what are now the Tonga–Fiji–Vanuatu–Solomon Islands. The oldest exposed rocks of Fiji (the Yavuna Group), found in western Viti Levu, are about 36–34 myr old (Hathway 1994; Rodda 1994; Crawford et al. 2003) and form part of a basaltic–andesitic volcanic arc suite (Vitiaz Arc) with a crustal thickness of nearly 30 km, which is comparable to that of continent margins (Colley 2009). Vanuatu incorporates arc-derived conglomeratic rocks dated to 23 Ma (Oligocene–Miocene) but some of the contained limestone and tholeiitic basalt clasts may be as old as the Eocene and are consistent with this region being part of the exposed Vitiaz Arc around 33 Ma (Crawford et al. 2003). Heads (2016, 2018) speculated that the Vanuatu–Fiji–Tonga island groups did not inherit the majority of their plants and animals from Australia or Asia, but that the biota developed as metapopulations that have survived and evolved on ephemeral islands of the ancient Vitiaz island arc system, possibly before the formation of the Pacific Ocean, until Fiji and associated archipelagos were formed. In contrast, molecular dating of the plant genus *Cyrtandra* (Gesneriaceae) indicates that the genus originated in Fiji around 9 Ma and that the archipelago played an important role in the evolution of the genus with onward dispersals, founder events, and diversifications in French Polynesia, Hawaii, and Vanuatu (Johnson et al. 2017). These views, or results, are mutually incompatible.

The Loyalty Rise, with several recently uplifted calcareous islands, is positioned between Grande Terre (the main island of New Caledonia) and Vanuatu. Heads (2018) stated that the biota of the Loyalty Islands is more similar to that of Vanuatu than to the geographically closer Grande Terre. He explained this by metapopulation vicariance, arguing that the Loyalty–Three Kings volcanic arc separated from the Vitiaz–Tonga–Kermadec Ridge around 50 Ma, followed by westward plate rollback, and that the Loyalty Islands came into juxtaposition with Grande Terre around 35 Ma (see Schellart et al. 2006). This biotic affinity has been demonstrated in crickets, but the divergence time was estimated to around 2 Ma (Nattier et al. 2011).

In a dating analysis of Pacific plants, Swenson et al. (2014) demonstrated that Sapotaceae progressively extended its distribution into the Pacific from west to east and that taxa are never older than the islands they inhabit except for those on the Hawaiian Islands. It was found that Oahu, a Hawaiian island that formed 2.6–3.0 Ma (Price and Clague 2002), is inhabited by *Planchonella sandwicensis* with one deep split at around 8.8 Ma (5.2–12.9 Ma), which predates the age of any of the current main Hawaiian Islands. It remains unclear whether this observation was an artifact of incomplete sampling, island-hopping, or metapopulation vicariance that stretches back to the Cretaceous sensu Heads (2018).

LDD and metapopulation vicariance offer incompatible perspectives on the origin of the Pacific biota. To explore this conflict, we have modeled the geographic movements of the plant genus *Planchonella* using a phylogeographic diffusion model implemented in BEAST (Lemey et al. 2010). *Planchonella* comprises 110 described species covering an area defined by Seychelles in the west, French Polynesia in the southeast, and Hawaii in the northeast (Swenson et al. 2013). The genus inhabits areas of diverse geological origin including continental landmasses (Australia/New Guinea), old uplifted continental terranes (New Caledonia), composite linear island arc systems (Vanuatu), isolated oceanic islands (Cook Islands), and hotspot island chains (Hawaiian Islands). We take particular interest in the following issues: (1) whether there is molecular evidence for metapopulation vicariance; (2) whether hotspot island chains are colonized in an order similar to that in which they develop; (3) whether species in Fiji and Vanuatu are closely related and have evolved *in situ*; (4) whether species in the Loyalty Islands are more closely related to those in Vanuatu or Grande Terre; and (5) whether *P. sandwicensis* is an old or a young lineage with respect to the Hawaiian Islands. These questions are summarized in five testable hypotheses (Table 1).

**Table 1.** Hypotheses (H$CDATA[$CDATA[$_{1}$$–H$CDATA[$CDATA[$_{5}$$) of colonization modes in the Pacific tested with *Planchonella* (Sapotaceae)

Hypothesis	Expected observation
H$CDATA[$CDATA[$_{1}$$—Metapopulation vicariance with local ecological dispersal drives colonization of Pacific islands (Heads 2011, 2017, 2018).	- Species inhabiting oceanic islands are usually older than the subaerial history of the island.
H$CDATA[$CDATA[$_{2}$$—Lineages inhabiting hotspot island-chains are colonized according to the progression rule.	- Species or populations progressively colonize younger islands and splits are consistent with the subaerial history of the island.
H$CDATA[$CDATA[$_{3}$$—Species in Fiji and Vanuatu have evolved *in situ*, not being derived from congeners in Australasia or Malesia (Heads 2006).	- Species from Fiji and Vanuatu are genetically related, form a monophyletic group, and not derived from Australasian ancestors.
H$CDATA[$CDATA[$_{4}$$—Biological similarity between the Loyalty Islands and Vanuatu is evidence of metapopulation vicariance due to westward rollback of the Loyalty Island ridge (Heads 2018).	- Accessions from the Loyalty Islands are more closely related to accessions from Vanuatu islands than to New Caledonia and stem from at least 30 Ma.
H$CDATA[$CDATA[$_{5}$$—*Planchonella sandwicensis* in Hawaii is an old lineage established by metapopulation vicariance sensu Heads (2018).	- Splits among Hawaiian accessions are much older than any of the existing islands, and do not conform with the progression rule.

## Materials and Methods

### Classification and Sampling

**Figure 1.**
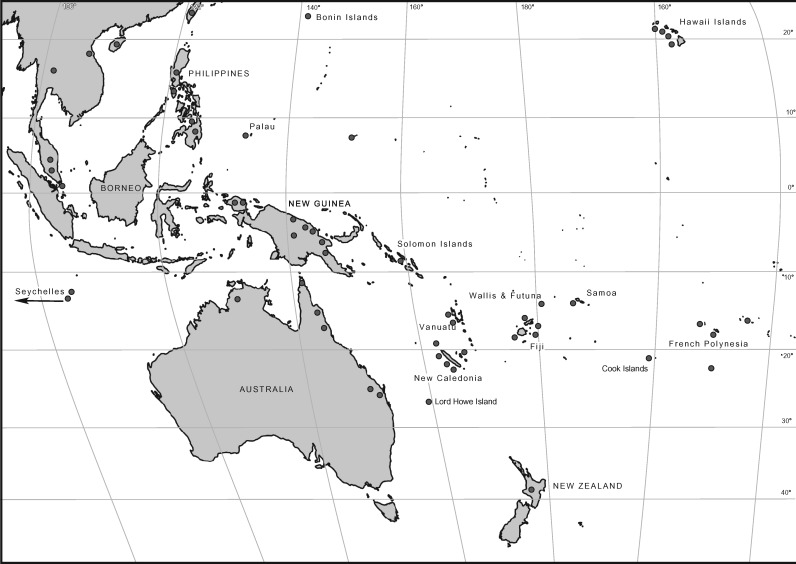
Map of Southeast Asia and the Pacific, shown in Mollweide projection, indicating where species of *Planchonella* (plant family Sapotaceae) were sampled. Dots may represent more than one accession and in some cases are placed adjacent to the island to avoid shadowing the landmass.

*Planchonella*, subfamily Chrysophylloideae, includes around 110 species (Swenson et al. 2013). We sampled 144 accessions, representing 80 recognized species across the range of the genus (Fig. 1). Most unavailable species are from New Guinea and the Solomon Islands. When possible, widespread species were sampled from multiple areas. *Planchonella obovata*, the generic type, is sampled from eight areas. *Planchonella sandwicensis*, endemic to Hawaii, is represented by 20 accessions from all islands it occupies in order to capture available genetic diversity. Material from the Bonin, Caroline, Cook, Samoa, and Solomon Islands in the Pacific, and the Seychelles in the Indian Ocean, are represented for the first time (Table 2). The phylogeny was rooted on *Pleioluma*, sister to the rest of the subfamily (except *Donella*) in Australasia (Swenson et al. 2013, 2014). The outgroup also includes members of *Amorphospermum, Magodendron, Van-royena*, three species of *Niemeyera* and all type species of the six subgenera of *Pycnandra* in order to have a comprehensive outgroup in case any of the ingroup accessions were found not to be members of *Planchonella*.

**Table 2.** Geographic areas (in alphabetic order), number of described *Planchonella* species, number of sampled species, and number of included samples

Geographic	Described	Sampled	Samples
area	species	species	included
Australia	15	14	16
Bonin Island	1	1	1
Caroline Islands	3	3	2
China (Hainan)	3	1	1
Cook Islands	1	1	1
Fiji	11	9	8
French Polynesia	1	1	5
Hawaii Islands	1	1	20
Hawaii	1	1	3
Kauai	1	1	4
Lanai	1	1	1
Oahu	1	1	7
Maui	1	1	3
Molokai	1	1	2
Malaysia (cultivated in Singapore)	6	4	4
New Caledonia	37	37	42
New Guinea	27	18	16
Philippines	8	5	4
Samoa	4	4	3
Seychelles	1	1	2
Solomon Islands	9	6	1
Taiwan	2	2	1
Thailand	4	4	3
Vanuatu	4	3	3
Vietnam	3	1	1
Wallis and Futuna	4	4	5

### Molecular Data and Analyses

We selected and adapted the data set of nuclear ribosomal DNA (ETS, ITS), the nuclear gene *RPB2*, and gap information coded as binary characters used by Swenson et al. (2013). We chose to focus on nuclear DNA because chloroplast markers lack sufficient phylogenetic information at this taxonomic level in Sapotaceae (Swenson et al. 2013). We modified the data set by excluding distant outgroups, while expanding the taxon sampling of interest in the ingroup. Extraction, amplification, and primers followed the protocols established by Bartish et al. (2005) for ITS, Swenson et al. (2008) for ETS, and Swenson et al. (2013) for *RPB2*. Molecular dating and phylogeographic diffusion analysis were performed in an integrated analysis using BEAST v1.8.4 (Drummond et al. 2012). The data (ETS, ITS, *RPB2*, gaps) was partitioned with a unique clock and substitution model for each aligned locus, while linking the tree model across all partitions. Each partition was tested for available substitution models in BEAST using jModeltest (Darriba et al. 2012), which assigned an HKY+G substitution model, and a simple model without gamma for the binary data, all with estimated base frequencies. Clock models for all partitions were set to uncorrelated lognormal distributions to allow different substitution rates and patterns of among-lineage rate variation across loci, with rate priors set to weakly informative exponential distributions (offset 0, mean 0.5), and with the default priors for the standard deviation of branch rates. We used a birth–death tree prior with default uninformative uniform priors for birth and death rates. Posterior probability (PP) of 0.95 or more is recognized as strong node support, 0.85–0.94 as weak, and values below 0.85 are not reported. Age estimates are reported with 95% highest posterior density (HPD) intervals. Taxa, vouchers, and GenBank accession numbers, together with a complete, aligned, and partitioned data matrix, are available from the Dryad Digital Repository: http://dx.doi.org/10.5061/dryad.p2gs002 (Supplementary Appendices S1 and S2).

### Node Calibration

Two nodes were temporally constrained, one using fossils and the other by applying a secondary calibration. Fossils of *Planchonella* are scarce and, apart from subfossils from the Marquesas Islands (Huebert and Allen 2016), are known only from New Zealand. These include a leaf impression from Oligocene–Miocene transition strata at Landslip Hill (Campbell 2002) and unpublished microfossils plus a flower bud containing pollen from Foulden Maar near Middlemarch, Otago (Swenson et al. 2014). Foulden Maar once hosted a rich mesothermal Lauraceae-dominated evergreen forest (Bannister et al. 2012) dated to 23.2 $CDATA[$CDATA[$\pm $$ 0.2 Ma (Lindqvist and Lee 2009; Lee et al. 2016). Fossils from these localities are allied to *Planchonella costata*, the only extant representative in New Zealand, which is sister to the Australian species *P. eerwah* (Swenson et al. 2013). Based on these being sister species, having rather long evolutionary histories, being descendants from a most recent common ancestor positioned on a short molecular branch, and a fossil that is very precisely dated, we argue that the fossil does not significantly pre-date the most recent common ancestor of *P. costata* and *P. eerwah* (node 37, Fig. 4). Hence, we have applied a fairly informative constraint on node 38 with an offset of 23.2 Ma with variable exponential decays of 0.055, 0.55, and 2.25, respectively, varying the upper 97.5% limit of the probability density. Hence, three different prior distributions were used, one of 23.2–23.4 Ma, one of 23.2–25.2 Ma, and one of 23.2–31.5 Ma. This approach was designed to compensate for the risk of erroneous dating of the fossil and a possible earlier arrival of *Planchonella* to New Zealand. Secondary calibration information can be used if fossils are scarce or absent (Ho and Philipps 2009; Ho et al. 2015). We used this to suggest a root age of *Pleioluma* approximated from the corresponding node of an earlier study (Swenson et al. 2014), modeled by a normal distribution (mean 48.6 Ma, SD 5.1) with 97.5% of the probability density between 38.6 and 58.6 Ma. A normal distribution is preferred over a uniform prior, because it does not impose unjustified hard boundaries on the potential node age.

### Phylogeographic Diffusion Analysis

Ancestral areas were estimated by modeling geographic movements across a continuous landscape using a relaxed random walk (Lemey et al. 2010), where locations are defined as coordinates in decimal grades for each accession. Random walk methods are preferable over the use of discrete areas, since they circumvent the need to define arbitrary operational areas, and this approach has been used on species-level phylogenies and is largely congruent with results from discrete methods (Nylinder et al. 2014, 2016; Bengtson et al. 2015; Johansson et al. 2018). Therefore, we argue that the biogeographic history of a plant genus scattered across numerous islands in the Pacific and adjacent regions, is better captured by a method that does not restrict the analysis to movement between larger, simplified discrete areas.

Accessions sampled from nearby locations (relevant for New Caledonia) could possibly cause problems in the likelihood calculation for the relaxed random walk, a problem solved by using a default jitter prior. The standard deviation on the random walk rate distribution was assigned an exponential prior with offset 0 and mean of 5.0. Such a prior is weak, but will potentially punish large rate changes associated with dispersal across large water bodies.

The phylogeographic diffusion analysis was run in BEAST v1.8.4 (Drummond et al. 2012) five times for 50 million generations each, sampling from the posterior every 25,000 generations. After removal of a portion of each run as burn-in, the chains were checked for convergence and stable state in Tracer v1.6 (Rambaut and Drummond 2004). Samples were then combined in LogCombiner and the trees were summarized in TreeAnnotator (part of the BEAST package) and visualized in FigTree v1.4.3 (Rambaut 2013). The continuous diffusion results were visualized in SpreaD3 (Bielejec et al. 2016) with HPD level of 80% for sampled geographic locations and visualized in Google Earth. Ancestral areas associated with nodes supported by PP $CDATA[$CDATA[$<$$0.95 were deleted in manual post-processing of the KML file from Google Earth. A raw KML file is available on Dryad as Supplementary Appendix S3.

## Results

### Maximum Clade Credibility Tree

**Figure 2.**
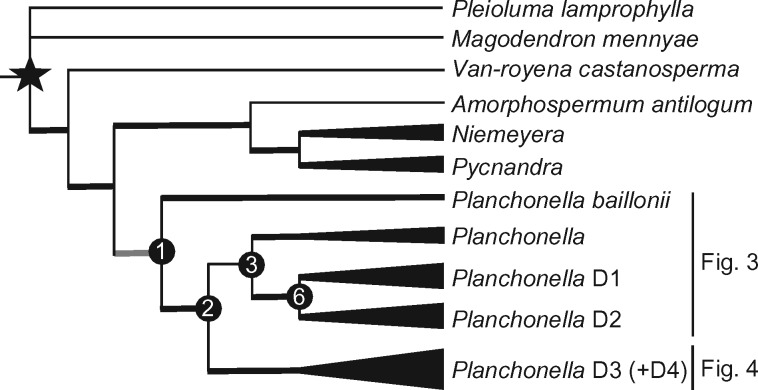
Phylogenetic overview of the plant group analyzed in this study, including the outgroup (*Pleioluma*), extended outgroup, and the ingroup *Planchonella* (Sapotaceae). Star represents the secondary calibrated node. Deep nodes of major clades are numbered in accordance with Figures 3 and 4.

**Figure 3.**
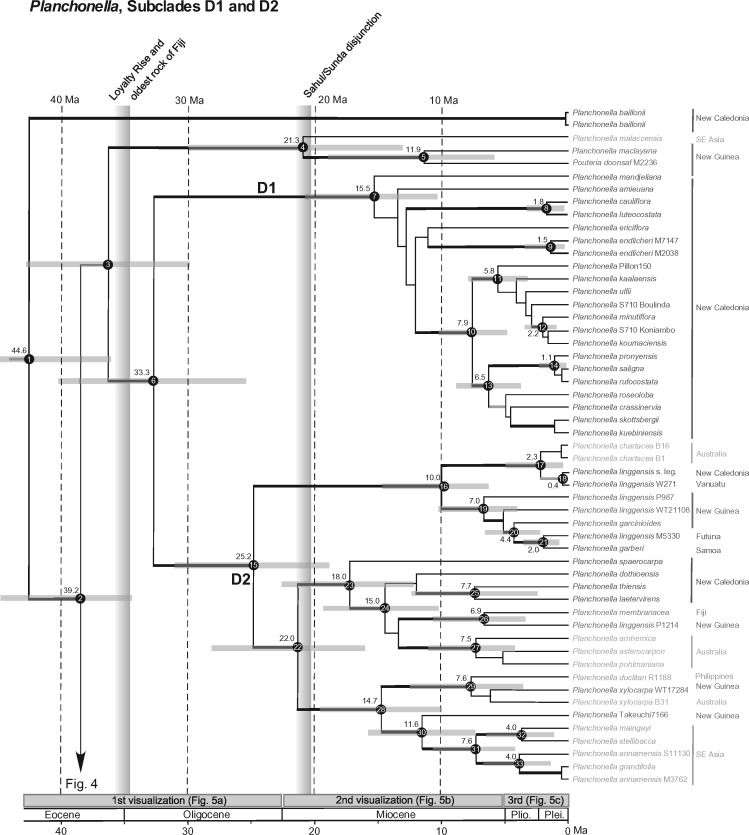
Chronogram of *Planchonella* (plant family Sapotaceae) subclades D1 and D2 (see Fig. 4 for subclades D3 and D4) distributed in Asia and the Pacific region (outgroup pruned away) obtained from BEAST analyses with median ages and 95% highest posterior density (HPD) intervals (blue horizontal bars). Clade support is shown as thick black branches (PP $CDATA[$CDATA[$ \ge $$ 0.95) or thick gray branches (PP 0.85–0.94). Numbered nodes and their estimated ages for three calibration schemes are found in Supplementary Appendix S4 available on Dryad. Vertical bars represent the time at which the Loyalty Rise came into juxtaposition with Grande Terre, which is the same age of the oldest subaerial rocks of Fiji, and the onset of the floristic exchange between the Sahul and Sunda shelves (Crayn et al. 2015). The three horizontal boxes above the geological scale indicate the selected visualizations of range expansion illustrated in Figure 5. Taxon names with vouchers represent multiple accessions. Terminals are color-coded by area: Asia (light green), Australia (orange), New Caledonia (blue), New Guinea (dark green), and Pacific Islands (red) apart from Fiji (purple).

**Figure 4.**
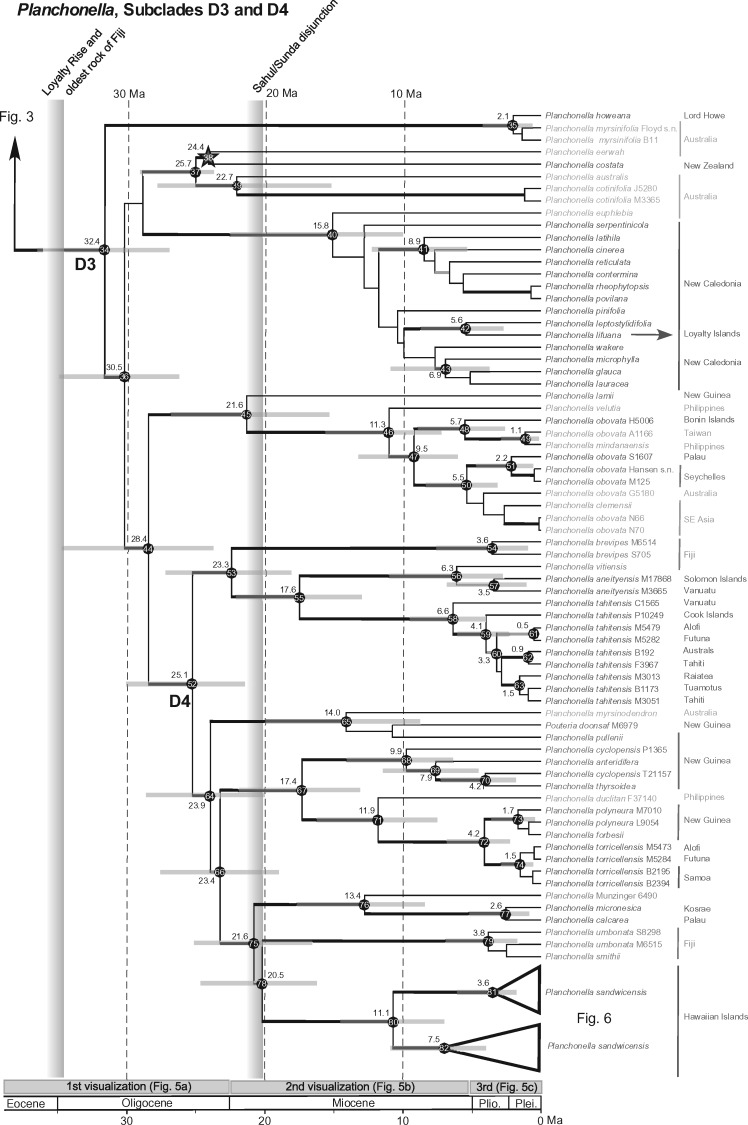
Continuation of the *Planchonella* (plant family Sapotaceae) chronogram as indicated in Figure 3, showing subclades D3 and D4. Details of the figure follow those of Figure 3, except that accessions from the Seychelles are color-coded in brown. The star points to the 23.2 myr fossil used for calibration. Accessions of the Hawaiian species *P. sandwicensis* are shown as two triangles, developed in Figure 6.

The overall phylogeny of Chrysophylloideae in Asia and Oceania matches the phylogenies reported by (Swenson et al., 2013, 2014), that is, *Planchonella baillonii* is the sister lineage to the rest of the genus, whose members are placed in three subclades (D1, D2, and D3). Several New Guinean and Pacific accessions are grouped in a strongly supported subclade of D3, here called D4 (Fig. 2). Nodes are numbered in each maximum clade credibility tree: nodes 1–33 in Figure 3 (subclades D1, D2) and nodes 34–82 in Figure 4 (subclades D3, D4). We choose to report the age estimates based on our calibration density of 23.2–31.5 Ma in order to capture as much uncertainty as possible. Estimated ages for all three calibration schemes are reported in Supplementary Appendix S4 available on Dryad. The maximum clade credibility tree is strongly supported overall except for weak relationships in clades that underwent rapid speciation in New Caledonia.

Several taxonomic problems are uncovered by the analysis. *Pouteria doonsaf* from New Guinea, represented by two accessions, is a member of *Planchonella*, but poorly circumscribed, since one accession groups with *P. maclayana* (node 5, Fig. 3) and the other with taxa from the Sunda Shelf (see node 65, Fig. 4). A similar result is revealed for the two accessions of *P. duclitan* from the Philippines, one placed in subclade D2 (node 29) and the other in subclade D3 (node 71). *Planchonella linggensis* and *P. chartacea* occur in different habitats, but form a mixed complex with *P. garcinioides* and *P. garberi* (subclade D2). The generic type (*P. obovata*) is widespread, ranging from the Seychelles in the west, Australia in the south, to the Bonin Islands in the east. All eight accessions represented in this study are placed in subclade D3, but mixed with *P. clemensii* and *P. mindanaensis*. Other polyphyletic species (*P. annamensis, P. cyclopensis*, and *P. polyneura*) pose additional challenges that are not resolvable here and need to be addressed elsewhere.

Divergence times in *Planchonella*, including the first generic split and crown nodes of subclades D1, D2, D3, and D4, are very close to previous estimates despite a more inclusive group with a greatly expanded sample set (80%). For example, the estimated median ages of the crown nodes of D2 and D4, respectively, are 25.2 Ma (18.6–32.2 Ma) and 25.6 Ma (21.1–31.3 Ma), which are close to the previous estimates of 25.0 Ma (18.9–31.2 Ma) and 24.0 Ma (19.7–29.0 Ma) (Swenson et al. 2014).

### Estimated Dates and Phylogeographic Diffusion Analysis

**Figure 5.**
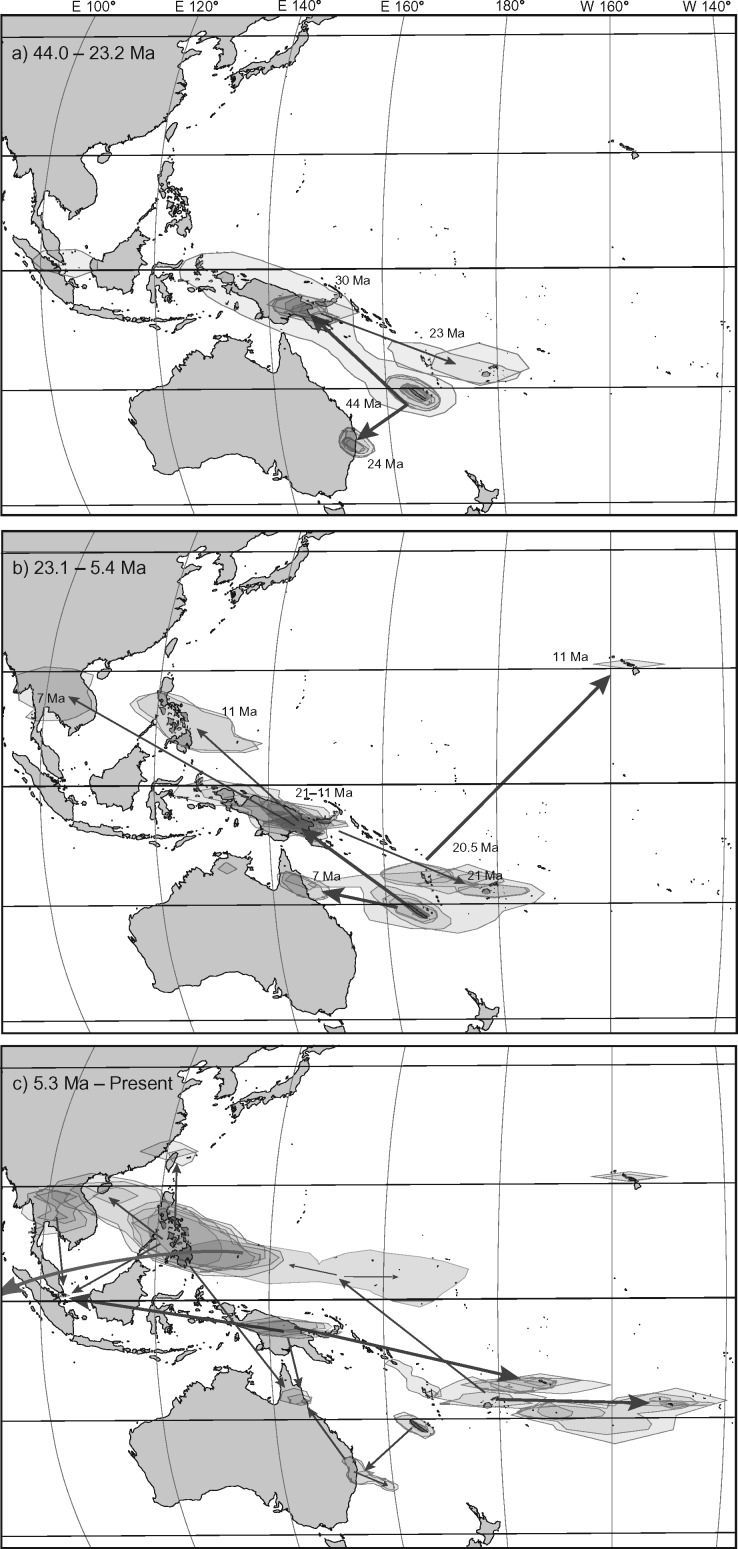
Visualization of range expansion in three time windows derived from the phylogeographic diffusion analysis with an HPD threshold level of 80%. a) Mid-Eocene and Oligocene, b) Miocene, c) Pliocene to present. Approximate ages of first colonization are indicated near the areas. Arrows represents old (thick) and more recent (thin) events in the respective time window. Red arrow represents a long-distance dispersal from Palau to the Seychelles at 2.2 Ma (0.4–4.8 Ma).

The phylogeographic diffusion analysis estimates the ancestral area of *Planchonella* as being in the Sahul Shelf area, while the genus originated in the region of New Caledonia at 44–46 Ma (36.6–54.2 Ma). This origin was probably not on present-day New Caledonia proper, which became subaerial around 37 Ma (Grandcolas et al. 2008; Swenson et al. 2014). In the interval 30–24 Ma, lineages back-dispersed to New Guinea and Australia, and from New Guinea expanded into the Pacific at 23 Ma (Fig. 5a). From 23 to about 7 Ma, the genus radiated across New Caledonia, Fiji, and Southeast Asia (Fig. 5b). From 5.3 Ma to the present, colonization occurred in almost all directions with the more recent lineages being present on younger oceanic islands (Fig. 5c). New Caledonia excluded, oceanic islands in the Pacific have been colonized at least 27 times. The deepest splits among Pacific island taxa are found in subclade D4 and are between 23.4 Ma (18.9–28.9 Ma, node 66) and 23.3 Ma (18.3–29.0 Ma, node 53). Fiji seems to have constituted an important landmass for range expansion. The Fijian species do not form a monophyletic group; instead, *Planchonella* has colonized Fiji three to five times. Accessions collected in Fiji and Vanuatu are never sisters. *Planchonella lifuana*, a species confined to the Loyalty Islands, split from its sister species, *P. leptostylidifolia*, around 5.6 Ma (2.9–8.7 Ma; Fig. 4).

**Figure 6.**
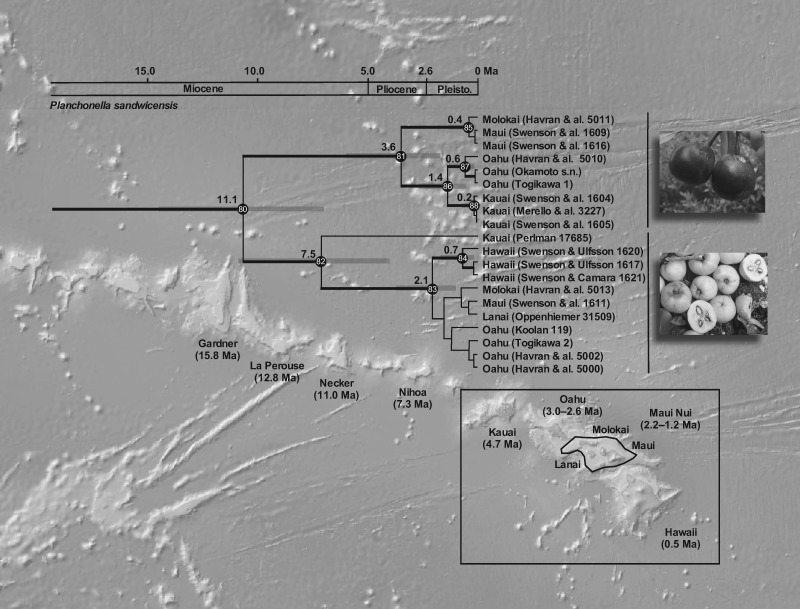
Chronogram of the Hawaiian endemic species *Planchonella sandwicensis* using 20 accessions (island: voucher) visualized on a topographic relief base-map of the Hawaiian Ridge and the Hawaiian Islands (National Oceanic and Atmospheric Administration: maps.ngdc.noaa.gov). Subaerial islands are shown in the rectangle and Maui Nui, with a maximum extent around 1.2 Ma, is encircled. Ages of islands and seamounts follow Price (2004). The lineage splits at 11.1 Ma (7.3–15.5 Ma), leading to purple- and yellow-fruited clades. Supported nodes are numbered and age estimates are found in Supplementary Appendix S4 available on Dryad (bathymetric map obtained from: NOAA National Centers for Environmental Information).

A divergence event in the area of Fiji 20.5 Ma (15.6–25.8 Ma) led to one lineage becoming established in the Hawaiian–Emperor seamount chain in which a subsequent split occurred at 11.1 Ma (7.43–15.5 Ma). The lineage present in Hawaii today is recognized as *Planchonella sandwicensis* but constitutes two strongly supported lineages with contrasting fruit colors: dark purple and yellow (Fig. 6). The crown node of the purple-fruited lineage is estimated at 3.6 Ma (1.7–6.2 Ma), whereas that of the yellow-fruited lineage is at 7.5 Ma (4.1–11.5 Ma). Other splits with phylogenetic support are younger than or of the same age as the islands that they inhabit. All samples from the same island of either the purple- (strong support) or yellow-fruited lineage (strong or no support) group according to the island they occupy, provided that we recognize Maui Nui, a paleo-island incorporating Kahoolawe, Lanai, Maui, and Molokai that reached its maximum size at 1.2 Ma (Price 2004).

## Discussion

### Metapopulation Vicariance and Progression Rule (H1, H2)

Our molecular dating of *Planchonella* in the Pacific region shows that the origins of island taxa are consistent with island ages, which is a crucial observation for testing the idea of metapopulation vicariance (Heads 2011, 2017, 2018). This is an important issue in itself but also bears on the question of whether the ages of oceanic islands can be used for setting temporal constraints in divergence-time analyses. Swenson et al. (2014) explored the effect of molecular and geological calibration (one type of temporal constraint) in their dating analyses of Chrysophylloideae in Australasia and the Pacific. Metapopulation vicariance was not supported in that study and the median ages of splits were never inferred to be older than the island(s) they inhabit, with the exception of the Hawaiian Islands, an issue discussed below. In this study of *Planchonella*, we include accessions from the Bonin, Caroline, Cook, Samoa, and Solomon Islands to further test the hypothesis of metapopulation vicariance.

The Bonin or Ogasawara Islands, located about 1000 km south of Tokyo, are up to 50 myr old and originated from an extensive submarine arc system that developed east of the Philippine Sea Plate (Hall 2002). Islands of this system have possibly been subaerial since 36 Ma (Kanayama et al. 2014) and might have harbored Sapotaceae members for considerable periods. However, our analyses show the contrary. *Planchonella obovata* and its ancestor are present on the island at 5.7 Ma (2.8–9.3 Ma; node 48, Fig. 4) where they subsequently gave rise to sister populations in the Philippines and Taiwan—landmasses that have never been in contact (Hall 2002) and that are located 2100–2500 km west of the Bonin Islands.

The Caroline Islands are represented by Kosrae in the east and Palau in the west. Kosrae is located around 3140 km east of Palau, is about 630 m high, and 110 km$CDATA[$CDATA[$^{2}$$ in area. A hotspot volcanic chain formed this island 1.4–2.6 Ma (Keating et al. 1984). Palau consists in part of old island arc volcanoes that date to 20.1–37.7 Ma (Kobayashi 2000). During the middle Oligocene, these rocks were submerged and overlain by limestone but uplifted in the Miocene to an altitude of 220 m (Neall and Trewick 2008). Palau is clearly older than Kosrae and may have offered terrestrial habitats throughout the past 15 myr. These two islands harbor, respectively, the endemic species *Planchonella calcarea* and *P. micronesica*, here shown to be sisters (node 77, Fig. 4). Their divergence is estimated at 2.6 Ma (0.8–5.4 Ma) and is consistent with the islands’ ages, but the uncertainty level stretches beyond the origin of Kosrae. The stem age of this pair is estimated at 13.4 Ma (8.3–18.4 Ma) and, again, is consistent with the possible subaerial exposure of Palau. Hence, a likely scenario is that Palau was colonized first, with a subsequent colonization of Kosrae when that island formed 1.4–2.6 Ma.

The Cook Islands lie in the heart of the tropical South Pacific and form a long linear island chain with the Austral Islands in the east. The Cook Islands are divided into the Northern (not relevant here) and the Southern Group, the ages of which do not fit within a single hotspot model (Hoffmann 2009). *Planchonella tahitensis* occurs on three volcanic islands: Mangaia (17–20 Ma), Mitiaro (7–10 Ma), and Rarotonga (2.3 Ma), which formed much later than the other islands (Hoffmann 2009). These three volcanic islands form more or less a triangle, separated, counter-clockwise from Mangaia, by 230, 260, and 200 km, and surrounded by waters $CDATA[$CDATA[$>$$2400 m deep, and have never been in contact with each other. The stem and crown age of *P. tahitensis* from Rarotonga are estimated at 6.6 (3.9–10.1) Ma and 4.1 (2.3–6.3) Ma (Fig. 4), which is much younger than the oldest parts of the Cook Islands. It would have been interesting to include accessions from all three islands to estimate population ages within the archipelago, but they were unavailable

The Samoan islands form another linear volcanic chain with the oldest seamounts in the west, the subaerial islands Savaii, Upolu, Tutuila, Manua Group in the center, and the active seamount Vailulu in the east. Savaii and Upolu are presently separated by shallow water and rest on the same shield-volcano edifice that began to develop around 5 Ma at depths of 3000–4000 m (McDougall 2010). Subaerial rocks of Upolu and Savaii are dated, respectively, to 1.4–2.8 Ma and 2 Ma, but the surface of Savaii is dominated by young (Pleistocene–Holocene) volcanic rocks. These islands are inhabited by *Planchonella garberi* and *P. torricellensis*, accessions placed in different parts of the phylogeny (node 21 in Fig. 3, node 74 in Fig. 4). The former is sister to one accession of *P. linggensis* from Futuna, located 930 km to the west, a split estimated at 2.0 Ma (0.8–3.9 Ma). The latter is represented by four accessions, one each from Alofi, Futuna, Savaii, and Upolu, two sister pairs estimated to have diverged 1.5 Ma (0.5–2.9 Ma). Again, splits between populations are young and consistent with the host islands’ ages.

The Solomon Islands constitute an archipelago of more than 1000 generally mountainous islands positioned between the Makira Trench to the southwest and the Solomon Trench to the northeast. The islands are not strictly oceanic in origin but rather have a composite geological history from the Cretaceous to the present (Davies 2009). This makes them prime candidates to harbor old lineages of Sapotaceae. About ten species of *Planchonella* are known from the archipelago, but material of only one species, *P. aneityensis*, yielded molecular data. *Planchonella aneityensis* is distributed on both Santa Ana Island (Solomon Islands) and Espiritu Santo (Vanuatu). A split within this species is dated to about 3.5 Ma (node 57) but is without phylogenetic support (Fig. 4). This species is sister to *P. vitiensis* from Fiji, having a common ancestry estimated to 6.3 Ma (2.7–11.2 Ma) and not older than any of their host islands.

In summary, the inferred phylogenetic relationships and molecular dates of *Planchonella* inhabiting the non-hotspot or hotspot islands of the Bonin, Caroline, Cook, Samoa, and Solomon groups show that they are derived lineages, consistent with the age of the islands they inhabit (Fig. 5c). In other words, even when we assumed a large amount of uncertainty in our calibration, our analysis finds no support for metapopulation vicariance (H$CDATA[$CDATA[$_{1})$$ in *Planchonella*. Instead, our results support a scenario whereby islands are colonized in subsequent order after their emergence (H$CDATA[$CDATA[$_{2})$$, strengthening the observation by Swenson et al. (2014) and rejecting the idea of metapopulation vicariance (Table 1).

### The Seychelles

North of Madagascar in the Indian Ocean lie the Seychelles islands, which, in part, consist of Gondwanan continental fragments that reached their current position some 63–65 Ma (Frey 2009). Despite providing terrestrial habitats during much of the Cenozoic, the Seychelles have been colonized by dispersal, for example, by Rubiaceae from Africa, Asia, and Madagascar in several waves (Kainulainen et al. 2017). The Seychelles are the westernmost outpost of *Planchonella*, where the widespread species *P. obovata* occupies the two main islands Mahé and Praslin. Sister to these accessions is the population from Palau, located in a straight line 8900 km to the east, with a divergence time of roughly 2.2 Ma (0.4–4.8 Ma; node 51, Figs. 4, 5c). Disjunctions of this kind can be explained only by LDD, which has also been inferred in *Amaracarpus* (Rubiaceae; Razafimandimbison et al. 2017).

### Planchonella in Fiji and Vanuatu (H2, H3)

The Solomons–Vanuatu–Fiji–Tonga are all part of the Melanesian arc system that developed over the past 40–45 myr (Hall 2002; Crawford et al. 2003; Schellart et al. 2006). By the end of the Eocene (35 Ma), this arc system began to split owing to back-arc spreading in the South Fiji Basin associated with eastward rollback of the Pacific oceanic crustal slab, which eventually resulted in three separate trench systems: Vanuatu, Vitiaz, and Tonga. Around 8–12 Ma, back-arc spreading began to form the North Fiji Basin, causing progressive isolation and clockwise rotation of the Vanuatu segment, counter-clockwise rotation of the Fiji segment, and cessation of activity in the Vitiaz Trench. In short, during the past 45 myr, Vanuatu experienced tectonism and at least three major phases of arc-related volcanism that probably generated some terrestrial environments in the region since the Oligocene. Heads (2006, 2018) suggested that the Vanuatu–Fiji island arc probably inherited most of its biota from the old (Melanesian) arc, demonstrated by the endemic species being much older than any of the individual islands.

Our analyses include eight accessions from Fiji and three from Vanuatu, representing eight species (*Planchonella aneityensis P. brevipes, P. linggensis, P. membranacea*, $CDATA[$CDATA[$P$$. “Munzinger6490”, *P. smithii, P. umbonata*, and *P. vitiensis*). These species are placed in seven clades scattered across the phylogeny, two in Clade D2 (Fig. 3) and five in Clade D4 (Fig. 4). The oldest subaerial rocks in Fiji and Vanuatu are dated to around 36 and 23 Ma, respectively (Crawford et al. 2003). *Planchonella brevipes* from Fiji has an ancestral origin at 23.3 Ma (18.3–29.0 Ma) and *P. linggensis* from Vanuatu represents the most recent arrival in the area at only 0.4 Ma. Sister relationships of these accessions include New Caledonia/Vanuatu, New Guinea/Fiji (Viti Levu), Solomon Islands/Vanuatu, Micronesia/Fiji (Vanua Levu), and Hawaii/Fiji. The phylogeographic diffusion analysis suggests that this area was colonized twice from New Guinea, with subsequent splits at 23.3 Ma (18.3–29.0 Ma) and 21.6 Ma (17.1–27.0 Ma). The first event resulted in radiation in Vanuatu and Fiji at 6 Ma with an onward colonization of the Cook Islands and French Polynesia, all in line with the progression rule. The second lineage split in an area west of Vanuatu and formed the lineages now present in Fiji and the Hawaiian Islands. An additional lineage, possibly with an origin in New Caledonia, split in the middle Miocene and progressed into New Guinea and Fiji at 6.9 Ma (3.4–11.2 Ma; node 26). Hence, *Planchonella* has had representatives in Fiji and Vanuatu for the past 20 myr, derived twice from New Guinea, and possibly twice from New Caledonia. The area probably formed an important genetic pool in the Miocene for onward colonization of islands progressively developing in the Pacific, especially supporting the patterns reported for the Society Islands and Hawaii (Hembry and Balukjian 2016; Claridge et al. 2017), but these areas have also received recent immigrants. Our data and analyses reject hypotheses H$CDATA[$CDATA[$_{1}$$ and H$CDATA[$CDATA[$_{3}$$ that the flora is older than Vanuatu and Fiji, and that it evolved on the ancient Melanesian arc, but is reconcilable with H$CDATA[$CDATA[$_{2}$$ in that the lineages have colonized oceanic islands subsequent to their development (Table 1).

### Similarity Between the Loyalty Islands and Vanuatu (H4)

The presumed higher biological similarity between the Loyalty Islands and Vanuatu compared with the more closely located Grande Terre (New Caledonia) could be explained by metapopulation vicariance and westward rollback of the Loyalty Ridge from 50 to 35 Ma (Heads 2018). The Loyalty Islands are positioned on the Loyalty Ridge and derived from Oligocene volcanic seamounts capped by thick limestones (Bogdanov et al. 2007). Four of these seamounts have been uplifted during the past 2 myr to about 100 m (Lifou) and 130 m (Maré) above sea level. The islands are undoubtedly young based on their marine caprocks, but old lineages could have become established on the islands.

*Planchonella lifuana* is the only congener endemic to the Loyalty Islands where it grows on calcareous substrates. This species is embedded in a clade of 13 species endemic to New Caledonia and all except one are restricted to ultramafic substrates. *Planchonella lifuana* is sister to *P. leptostylidifolia* from which it split at 5.6 Ma (2.9–8.7 Ma; node 42, Fig. 4). The estimate is slightly older than the age of the Loyalties, but much closer to the island age than the 35–50 myr required in order to concur with ridge rollback and metapopulation vicariance. Another example from Sapotaceae is a yet undescribed species of *Pycnandra* ($CDATA[$CDATA[$P$$. Butaud 3343), endemic to the Loyalties and embedded among species from Grande Terre, with an age of about 2.5 Ma (Swenson et al. 2014, 2015). Other examples of dated disjunctions between these islands are the geckos *Bavayia crassicollis* and *B. cyclura* from, respectively, the Loyalties and Grande Terre, dated to some 2 Ma (Skipwith et al. 2016). There are also young age disjunctions between the Loyalties and Vanuatu, exemplified by the sister crickets *Lebinthus lifouensis* and *L. santoensis*, dated to 2.1 Ma, and the widespread species *Cardiodactylus novaeguineae* dated to 0.2 Ma (Nattier et al. 2011). It suffices to say that molecular evidence indicates that sisters and widespread taxa exist in any combination of Grande Terre, Loyalty Islands, and Vanuatu, and that many split during the Pleistocene, rejecting both H$CDATA[$CDATA[$_{1}$$ and H$CDATA[$CDATA[$_{4}$$ of metapopulation vicariance dependent on westward ridge rollback (Table 1).

### Hawaiian Islands (H2 and H5)

The Hawaiian–Emperor seamount chain comprises eight main islands and numerous atolls and seamounts extending from the young island Hawaii (0.5 Ma) to Kure Atoll (29.8 Ma) in the northwest (Price and Clague 2002). The islands are of hotspot origin and Kauai (4.7 Ma) is currently the oldest high island in the chain. Northwest of Kauai are the small islands of Nihoa (7.3 Ma) and Necker (11.0 Ma). Six islands (Kauai, Oahu, Maui, Molokai, Lanai, and Hawaii) are inhabited by the morphologically confusing species *Planchonella sandwicensis* (Wagner et al. 2002). The crown age for $CDATA[$CDATA[$P$$. *sandwicensis* is estimated at 11.1 myr (7.3–15.5 myr), which splits into two well-supported lineages with crown ages of 3.6 myr (1.7–6.2 myr) and 7.5 myr (4.1–11.5 myr; Fig. 6). Subsequent splits group the accessions according to island of origin, but some with very weak support. Accessions of the two lineages have, respectively, deep purple and yellow fruits and the split at 11.1 Ma corresponds to a speciation event, but it is unclear if additional species or subspecies are recognizable. Taxonomic revision of these lineages will be explored in a subsequent paper.

The first split in the entire Hawaiian lineage corresponds to the age of Necker Island (11.0 myr), whereas the crown age for the yellow-fruited clade corresponds to the age of Nihoa (7.3 myr). This lineage later split at 2.1 Ma (1.0–3.8 Ma) and colonized Hawaii at 0.7 Ma (0.1–1.7 Ma), which coincides with the development of that island. Maui Nui and Oahu were colonized around 1.5 Ma, concurring with the islands’ ages, but support is weak and the direction of dispersal is not established. The purple-fruited clade began to radiate at 3.6 Ma (1.7–6.2 Ma; Fig. 6). The divergence time between populations in Molokai and Maui is estimated at 0.4 Ma (0.0–1.4 Ma) and coincides with subsidence and isolation of Maui Nui into separate islands (Price 2004). The split between populations on Oahu and Kauai occurred at 1.4 Ma (0.5–2.8 Ma) and coincides with the islands’ ages, but the population on Kauai seems to be derived. The youngest island, Hawaii, has never been colonized by this clade. The yellow-fruited clade concurs in part with the progression rule, having a potential origin in Kauai and a progressive colonization of younger islands, whereas the purple-fruited clade does not, owing to inferred back-dispersal to older islands.

Splits in Hawaiian *Planchonella* are apparently older than the islands that presently hold forests (nodes 80, 82; Fig. 6) and the gap to the stem node spans myr. Whether an extinct ancestor has ever been present on yet older islands, such as Gardner (15.8 Ma), will remain unknown. During the formation of Kauai, it is possible that no other islands in the chain had peaks over 1000 m (Price and Clague 2002), suggesting that extant Hawaiian taxa in montane habitats may have had an origin after the development of Kauai or may have experienced a severe bottleneck if derived from a pre-Kauai island. Price and Clague (2002) explained that this bottleneck may not have been as severe for coastal and lowland taxa. *Planchonella sandwicensis* is distributed in various habitats, including relatively dry and humid windward forests. The yellow-fruited clade appears to be distributed predominantly in the drier, leeward sides of islands—regions that have many lowland and coastal ecological attributes. Therefore, it is probable that the ancestor of *P. sandwicensis* was established from a lowland population on a pre-Kauai island. Other Hawaiian angiosperms, including the Hawaiian lobeliads (Campanulaceae) (Givnish et al. 2009) and *Hillebrandia* (Begoniaceae) (Clement et al. 2004), have origins much older than the current main islands.

Our results show that the Hawaiian lineage of *Planchonella* is older than the existing high islands, that the first split is consistent with the age of the two eroded Necker and Gardner islands, and that the species is not of Cretaceous age. Nevertheless, the lineage must have thrived somewhere, since it split from the most recent common ancestor at 20 Ma. It may have survived by island-hopping along the Hawaiian–Emperor seamount chain. Hence, the history of *Planchonella* in Hawaii concurs with island-hopping, but not with metapopulation vicariance sensu Heads (2018). We thereby reject H$CDATA[$CDATA[$_{5}$$ but, in part, support H$CDATA[$CDATA[$_{2}$$ in that islands are progressively colonized by dispersals subsequent to their origin and becoming habitable (Table 1).

### Fruit, Seeds, and Dispersal Agents

Our results strongly favor one hypothesis, that *Planchonella* expanded its distribution across the Pacific by LDD. The fruit type and dispersal agents of the genus were, and are, undoubtedly important features. Species of Sapotaceae form canopy and understory trees and shrubs in a wide range of vegetation categories. The fruit is a one-to-many-seeded berry that is commonly rich in sweet pulp, covered by a leathery outer pericarp. Seeds vary in size and form and are covered by a hard glossy, smooth testa. Predation of fruit and seeds in the family has been little studied, but fruits are consumed by primates, bats, birds, lizards, and even fish that eat the pulp and either spit out the seeds or swallow them for subsequent dispersal (Pennington 1991; Lobova et al. 2009). Fruit of *Planchonella* in the Pacific region are known to be eaten and dispersed by flying foxes, pigeons, and skinks (Banack 1998; Hall and Richards 2000; Sadlier et al. 2014). In New Caledonia, the Imperial-pigeon (*Ducula goliath*) has been filmed swallowing fruit of *P. endlicheri* and is therefore considered to be an important dispersal agent in New Caledonia (Supplementary Appendix S5 available on Dryad).

## Conclusions

Molecular dating of *Planchonella* across its entire range consistently finds support that oceanic islands harbor species of similar or younger age compared with the islands they inhabit. Colonization of islands often follows a progression rule pattern, but not without exceptions, as demonstrated by back-dispersal from islands to continental landmasses, separate late arrivals, and island-hopping in the Hawaiian archipelago. *Planchonella* has a long evolutionary history in Fiji but, as far as is known, not in Vanuatu, which indicates that Fiji probably formed an important source area for subsequent colonization of the Pacific. The hypothesized westward rollback of the Loyalty Island ridge, causing vicariance between Vanuatu and the Loyalty Islands, finds no support from our analysis of *Planchonella*. Young sister-group relationships, from late Miocene almost to the present, between Grande Terre, the Loyalty Islands, and Vanuatu exist in all combinations. The Hawaiian Islands host a lineage that colonized and split into two species at 11.1 Ma, possibly on the 11-myr-old Necker Island, an island that today has almost succumbed to erosion and subsidence. Suffice it to say, the biology of *Planchonella* rejects metapopulation vicariance and we predict that this will also be the case for most other groups when analyzed in detail.

The question remains as to whether it is meaningful to use the ages of oceanic island as maximum ages in divergence-time analyses. We agree with Pillon and Buerki (2017) and urge careful consideration of this approach. It is inappropriate to calibrate stem nodes of known old endemic island taxa that are evidently older than the origin of the island on which they grow. For example, the paleoherb *Lactoris fernandeziana*, endemic to the Juan Fernandez Island Masatierra, is an inappropriate “calibration point”, even if it has implicitly been suggested (Heads 2011), because *Lactoris* has a fossil record of some 90 myr (Gamerro and Barreda 2008) as opposed to the 4-myr geological history of Masatierra (Stuessy et al. 1984). In contrast, age estimates of many clades confined to New Caledonia concur with the view that the territory re-emerged around 37 Ma (Swenson et al. 2014; Nattier et al. 2017). Hence, we support the view of Ho et al. (2015) that biogeographic calibrations (based on geological and/or climatic data) can provide useful information for divergence-time estimates, but that any such calibration must be balanced and well justified.

## Supplementary Material

Data available from the Dryad Digital Repository: http://dx.doi.org/10.5061/dryad.p2gs002.
